# Modeling the effect of ascites-induced compression on ovarian cancer multicellular aggregates

**DOI:** 10.1242/dmm.034199

**Published:** 2018-09-25

**Authors:** Yuliya Klymenko, Rebecca B. Wates, Holly Weiss-Bilka, Rachel Lombard, Yueying Liu, Leigh Campbell, Oleg Kim, Diane Wagner, Matthew J. Ravosa, M. Sharon Stack

**Affiliations:** 1 Department of Biological Sciences, University of Notre Dame, Notre Dame, IN 46556, USA; 2 Harper Cancer Research Institute, University of Notre Dame, Notre Dame, IN 46617, USA; 3 Department of Pathology and Laboratory Medicine, University of Kansas Medical Center, Kansas City, KS 66160, USA; 4 Department of Applied and Computational Mathematics and Statistics, University of Notre Dame, Notre Dame, IN 46556, USA; 5 Department of Mathematics, University of California, Riverside, CA 92521, USA; 6 Department of Mechanical and Energy Engineering, Indiana University-Purdue University Indianapolis, Indianapolis, IN 46202, USA; 7 Department of Chemistry and Biochemistry, University of Notre Dame, Notre Dame, IN 46556, USA

**Keywords:** Ovarian cancer, Metastasis, Multicellular aggregates, Ascites, Mechanobiology, Compression

## Abstract

Epithelial ovarian cancer (EOC) is the most lethal gynecological malignancy. EOC dissemination is predominantly via direct extension of cells and multicellular aggregates (MCAs) into the peritoneal cavity, which adhere to and induce retraction of peritoneal mesothelium and proliferate in the submesothelial matrix to generate metastatic lesions. Metastasis is facilitated by the accumulation of malignant ascites (500 ml to >2 l), resulting in physical discomfort and abdominal distension, and leading to poor prognosis. Although intraperitoneal fluid pressure is normally subatmospheric, an average intraperitoneal pressure of 30 cmH_2_O (22.1 mmHg) has been reported in women with EOC. In this study, to enable experimental evaluation of the impact of high intraperitoneal pressure on EOC progression, two new *in vitro* model systems were developed. Initial experiments evaluated EOC MCAs in pressure vessels connected to an Instron to apply short-term compressive force. A Flexcell Compression Plus system was then used to enable longer-term compression of MCAs in custom-designed hydrogel carriers. Results show changes in the expression of genes related to epithelial-mesenchymal transition as well as altered dispersal of compressed MCAs on collagen gels. These new model systems have utility for future analyses of compression-induced mechanotransduction and the resulting impact on cellular responses related to intraperitoneal metastatic dissemination.

This article has an associated First Person interview with the first authors of the paper.

## Abstract (precis)

**Summary:** Development of new model systems with which to evaluate cellular responses to compression enables experimental modeling of the intraperitoneal force environment of ovarian cancer patients with tense ascites.

## INTRODUCTION

The presence of malignant ascites, an excess of fluid in the intra-abdominal cavity as a consequence of peritoneal carcinomatosis, is usually considered a terminal (stage IV) event associated with poor survival in most neoplasms. The pathophysiology of ascites accumulation is multifactorial and includes enhanced fluid secretion by the tumor, poor reabsorption through tumor-obstructed lymphatic channels in the diaphragm and increased microvascular permeability (Adam and Adam, 2004; Becker et al., 2006; Garrison et al., 1987; Rosenberg, 2006). In epithelial ovarian carcinoma (EOC), however, direct intraperitoneal exfoliation of primary tumor cells, survival of tumor cells as non-adherent multicellular clusters and widely disseminated intraperitoneal spread cause early failure of lymphatic drainage channels. Together with enhanced vascular permeability, peritoneal accumulation of malignant transudate is often observed in EOC even at early stages (I-III) of the disease (Burleson et al., 2006; Adam and Adam, 2004; Byrne et al., 2003; Hudson et al., 2008; Howlader et al., 2011). Ovarian cancer is the leading cause of carcinomatous ascites in women, responsible for 36-38% of all ascites cases (Ayantunde and Parsons, 2007; Parsons et al., 1996; Malik et al., 1991). It has been demonstrated that ascites secondary to ovarian malignancies can be conditionally controlled by debulking surgery in combination with platinum-based chemotherapy, resulting in increased progression-free survival of up to 1 year (Parsons et al., 1996; https://seer.cancer.gov/archive/csr/1975_2009_pops09/). Additionally, effective ascites reduction with immunotherapeutic agents (Burges et al., 2007; Heiss et al., 2005), transforming growth factor-β (TGFβ) blockers (Liao et al., 2011), matrix metalloproteinase (MMP) inhibitors (Parsons et al., 1997) and vascular endothelial growth factor (VEGF) antagonists (Byrne et al., 2003; Hamilton et al., 2008) in preclinical and clinical studies are observed.

Microscopic evaluation of human EOC ascites fluids has revealed the presence of anchorage-independent metastatically competent multicellular aggregates (MCAs) that can adhere to the peritoneal mesothelial cell surface and submesothelial matrix proteins to initiate secondary lesions (Burleson et al., 2004, 2006; Klymenko et al., 2017a); however, the MCA as a metastatic unit remains poorly understood. MCA integrity is maintained by cadherins, calcium-dependent cell-cell adhesion molecules that preserve tissue architecture in normal epithelia (Klymenko et al., 2017c). Heterogeneous cadherin expression is a hallmark of EOC tumors, with cells expressing epithelial (E-) cadherin, mesenchymal neural (N-) cadherin, both E- and N-cadherin in the same tumor (‘mixed’ cadherin phenotype), and both E- and N-cadherin in the same cell (‘hybrid’ cadherin phenotype) (Hudson et al., 2008; Klymenko et al., 2017a,c). Although E-cadherin expression enables MCAs to avoid anoikis and contributes to chemotherapy resistance, N-cadherin expression predominates in peritoneally anchored metastatic lesions and confers a more invasive phenotype to EOC cells, suggesting that cadherin switching contributes to metastatic progression in EOC (Klymenko et al., 2017a,b,c; Hudson et al., 2008).

In women with EOC, accumulated malignant ascites ranging from 500 ml to >2 l commonly results in abdominal distension (Hu et al., 2005; Rudlowski et al., 2006). The vast majority of women with advanced EOC produce >500 ml of ascites; in contrast, the peritoneal cavity of disease-free women contains only 50-20 ml of serous exudate (Shen-Gunther and Mannel, 2002). Ascites is correlated with both intraperitoneal and retroperitoneal spread and is an independent adverse prognostic factor (Ayhan et al., 2007; Rahaman et al., 2004). Indeed, patients who present with ascites at primary diagnosis exhibit poor survival, even in early-stage EOC (Lenhard et al., 2009). The presence of large volumes of ascites at diagnosis significantly worsens prognosis, even when cytology is negative (Dembo et al., 1990; Chung and Kozuch, 2008), and is associated with failure to achieve optimal surgical debulking (Szender et al., 2017). Furthermore, normal intraperitoneal fluid pressure is subatmospheric (−5 mmHg) in mammals (Henriksen et al., 1980), but averages 11.2 mmHg in patients with cirrhotic ascites (range 3.2-22 mmHg). In women with EOC accompanied by tense ascites, an average intraperitoneal pressure of 30 cmH_2_O (22.1 mmHg) has been measured (Gotlieb et al., 1998). This is similar in magnitude to the high interstitial pressure measured in solid tumors (22 mmHg in cervical cancer, 19-33 mmHg in head and neck squamous cell carcinoma, 38 mMHg in renal cancer) (Esquis et al., 2006). Furthermore, increased intraperitoneal pressure generated via CO_2_ pneumoperitoneum is associated with increased incidence of abdominal metastasis in a murine EOC model (Matsuzaki et al., 2009). Together, these data indicate significant correlation between the presence of ascites, intraperitoneal pressure and EOC metastasis. In this study, we report the development of two unique model systems with which to examine the impact of altered peritoneal mechanobiology on EOC MCAs.

## RESULTS

Increased peritoneal fluid pressure results from the presence of tense ascites in women with EOC; however, the potential impact of ascites-induced compression of EOC MCAs has not been evaluated owing to a lack of appropriate model systems. Our initial attempts to address this question focused on *in vivo* approaches to mimic ascites accumulation and were based on intraperitoneal injection of buffered albumin solutions of high oncotic pressure to induce an ‘artificial ascites’ environment. This approach was not successful, however, as fluids were rapidly resorbed within 2 h (data not shown). We therefore re-directed our approach to the development of new *in vitro* models.

**Fig. 1.**
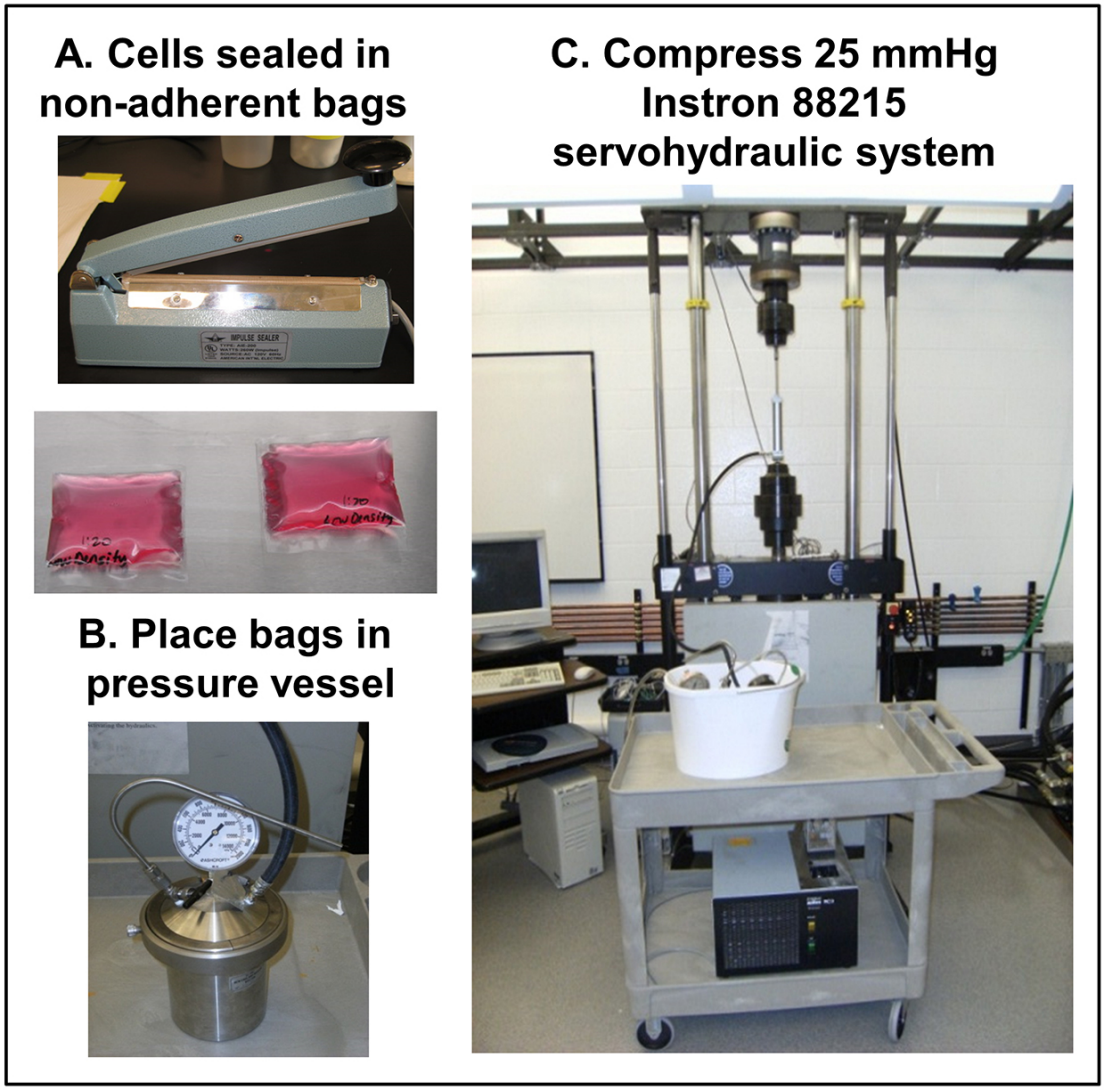
**Overview of EOC MCA compression via Instron.** (A) Cells (2×10^6^/ml; 12 ml) were seeded into non-adherent polyethylene bags, sealed and placed into an incubator overnight to enable MCA formation. (B) Cell-containing bags were transferred to pressure vessels pre-heated to 37°C. (C) Pressure was conveyed to the test vessel (0.5 pounds per square inch; 25 mmHg) using the Instron 88215 via displacement of water in a medium-duty hydraulic pump connected to the pressure vessel with stainless steel tubing. Two valves were closed to contain the fluid pressure. Temperature was maintained at 37°C with a water bath incubator. A control pressure vessel with cell-containing bags was sealed and incubated alongside the test pressure vessel, but was maintained at atmospheric pressure. After 8 h, bags were removed from the vessels and cells were harvested for analysis.

**Fig. 2.**
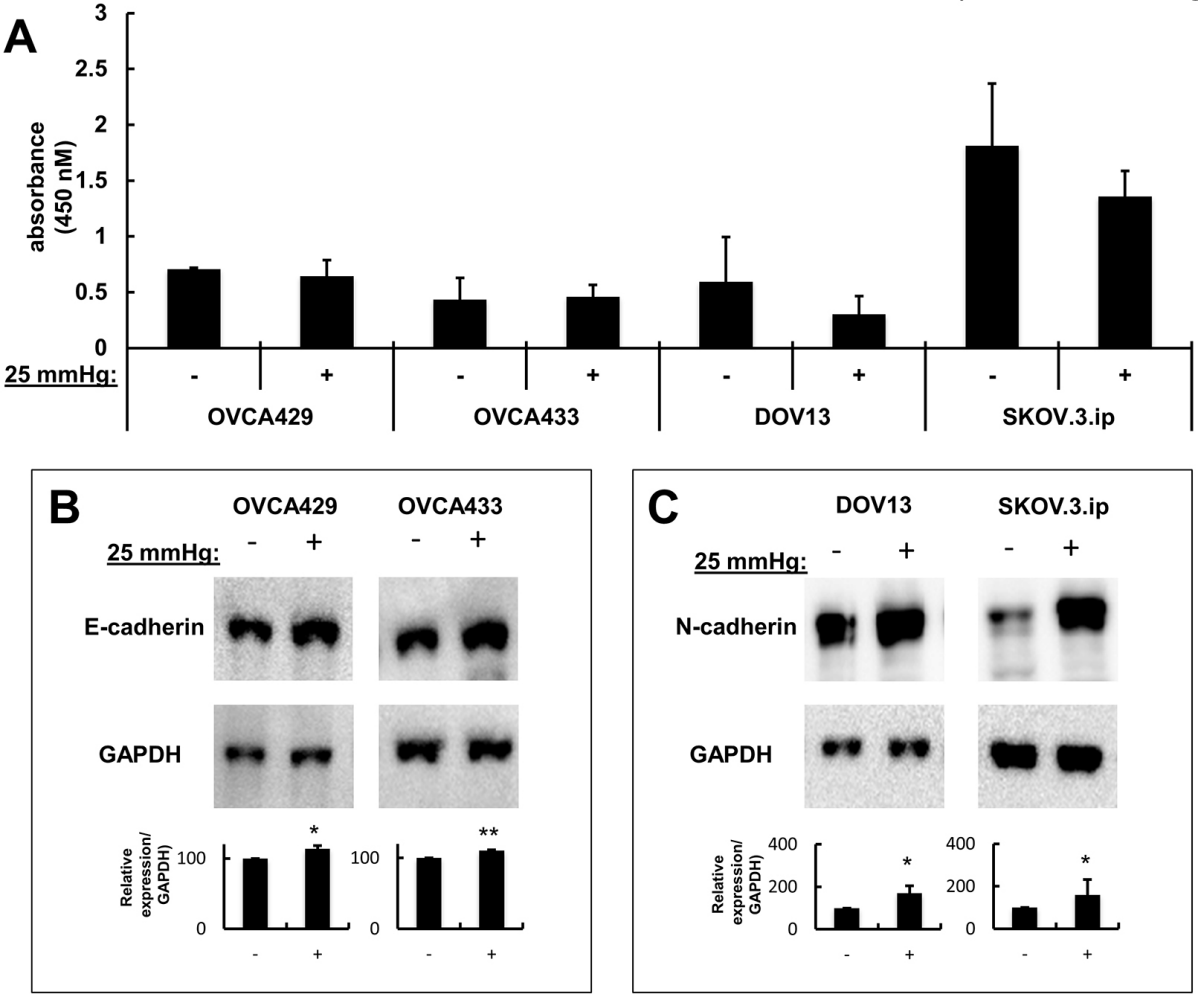
**Compression of MCAs in polyethylene bags alters cadherin expression but does not affect proliferation.** Cells were cultured as described in Fig. 1 in the absence (−) or presence (+) of compression (25 mmHg; 8 h). (A) Proliferation was evaluated using a Cell Proliferation ELISA (Sigma-Aldrich) according to the manufacturer's specifications. Assays were performed in triplicate and *P*-values were calculated by paired Student's *t*-test comparing compressed versus uncompressed cells. No significant changes in proliferation were observed. (B,C) Cell lysates were processed for western blotting to detect expression of E-cadherin (B) or N-cadherin (C) as described in the Materials and Methods. Experiments were repeated in biological triplicate. Antibodies used were rabbit monoclonal anti-E-cadherin (1:1000), mouse monoclonal anti-N-cadherin (1:1000), peroxidase-conjugated goat anti-mouse secondary antibody (1:4000) and mouse monoclonal anti-GAPDH (1:25,000). Densitometric analysis of western blot protein bands was performed in ImageJ. *P<0.05; **P<0.01.

In initial experiments using non-adherent polyethylene culture bags, EOC MCAs were sealed into fluid-filled bags and transferred to pressure vessels at 37°C (Fig. 1A,B). Pressure was conveyed at 25 mmHg to the test vessel using an Instron, as previously reported for chondrocytes (Fig. 1C) (Wagner et al., 2008; Steward et al., 2014). Control EOC MCAs were sealed and incubated in an identical pressure vessel that was temperature controlled and maintained at atmospheric pressure. After static compression for various time points, cells were harvested for evaluation. Overall, no significant changes in cellular proliferation were observed across four cell lines subjected to compression relative to uncompressed MCAs (Fig. 2A). As cadherins are important for the maintenance of MCA integrity, survival and metastatic dissemination (Klymenko et al., 2017a,b,c), the effect of compression on cellular cadherin expression profiles was examined. Four cell lines were evaluated, two of which express E-cadherin (OvCa429 and OvCa433) and two N-cadherin-expressing lines (DOV13 and SKOV.3.ip). A small but significant increase in E-cadherin expression was observed in OvCa429 and OvCa433 cells relative to uncompressed controls (Fig. 2B), while an approximately twofold change in N-cadherin was observed in compressed DOV13 and SKOV.3.ip cells (Fig. 2C). No gain of N-cadherin was observed in OvCa429 or OvCa433 cells, nor of E-cadherin in DOV13 or SKOV.3.ip cells, in response to 8 h compression (data not shown).

**Fig. 3.**
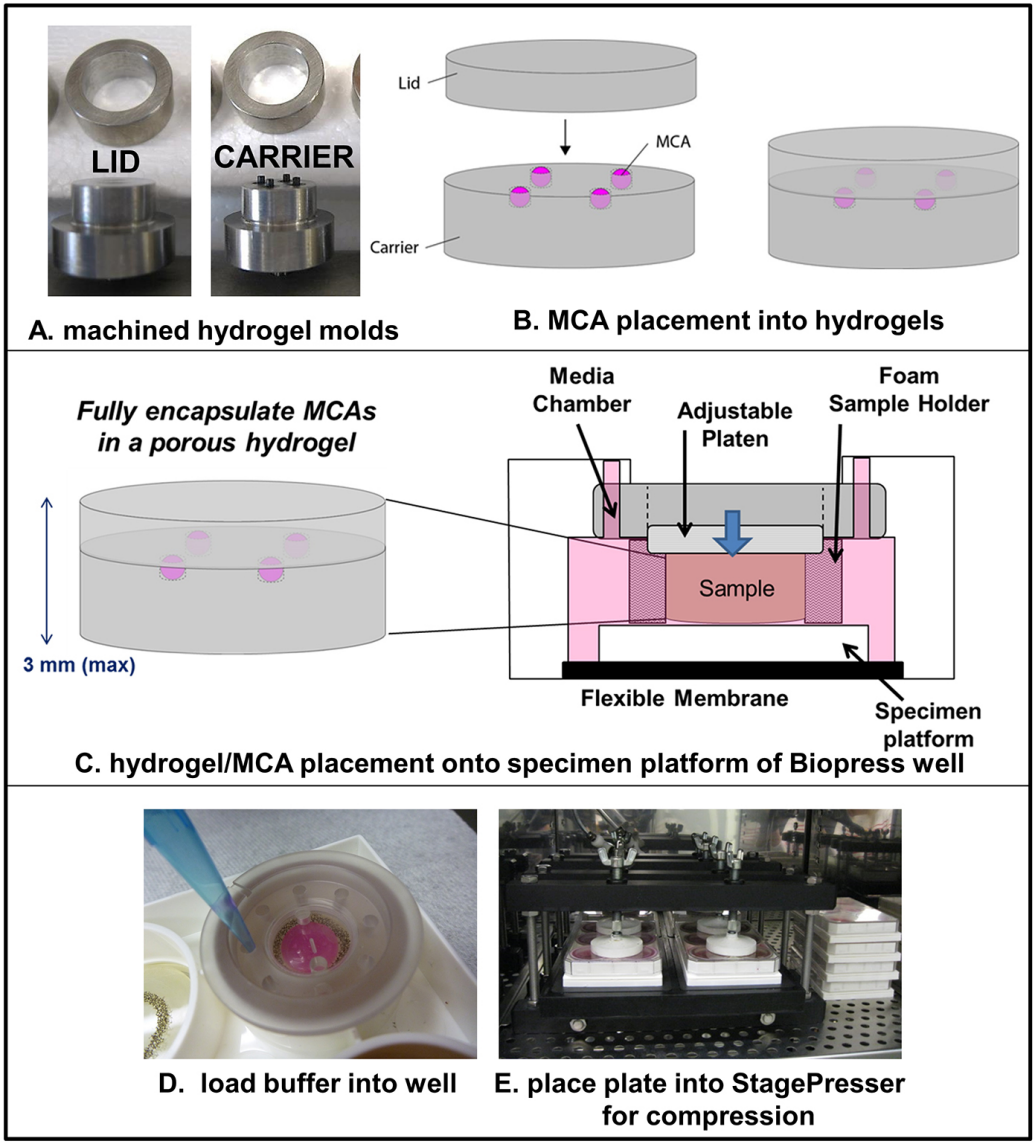
**Overview of EOC MCA compression via Flexcell Compression Plus.** (A) Two sets of metal molds were fabricated to generate ‘carrier’ and ‘lid’ hydrogels. (B) Diagram depicting MCA placement into hydrogel wells and hydrogel lid placement. (C) Diagram depicting hydrogel/MCA placement into BioPress plate well and interaction with the Flexcell Compression Plus platen. (D) Placement of hydrogel/MCAs into sample well of BioPress plate and addition of culture medium. (E) Assembly of four BioPress plates (containing a total of 24 hydrogels) into the Flexcell Compression Plus StagePresser. Following assembly, plates are subject to static compression at 3.18-3.53 kPa (∼24-26.5 mmHg) for 6 h or 24 h. On the right side of the photo are four control hydrogel/MCA-containing plates (containing a total of 24 control hydrogels) that are incubated under identical conditions but are not compressed.

Because gas exchange is less efficient in polyethylene culture bags, we adapted a Flexcell Compression Plus System placed in a tissue culture incubator to examine MCA compression at additional longer time points. To provide equivalent physical contact with the compression platens and thereby ensure even loading conditions, MCAs were placed in custom hydrogel carriers designed to fit within the BioPress culture plates. Briefly, custom molds were fabricated to enable production of ‘carrier’ and ‘lid’ hydrogels (Fig. 3A,B; Fig. S1). The carrier hydrogels were placed into BioPress culture plates and seeded with cells to enable MCA formation (Fig. S2). After applying the hydrogel lid and assembly of the Compression Plus apparatus, MCAs were compressed at ∼25 mmHg for 6 h or 24 h prior to analysis (Fig. 3C-E). Control MCAs were prepared identically and placed in BioPress plates in the same incubator, but were not compressed.

**Fig. 4.**
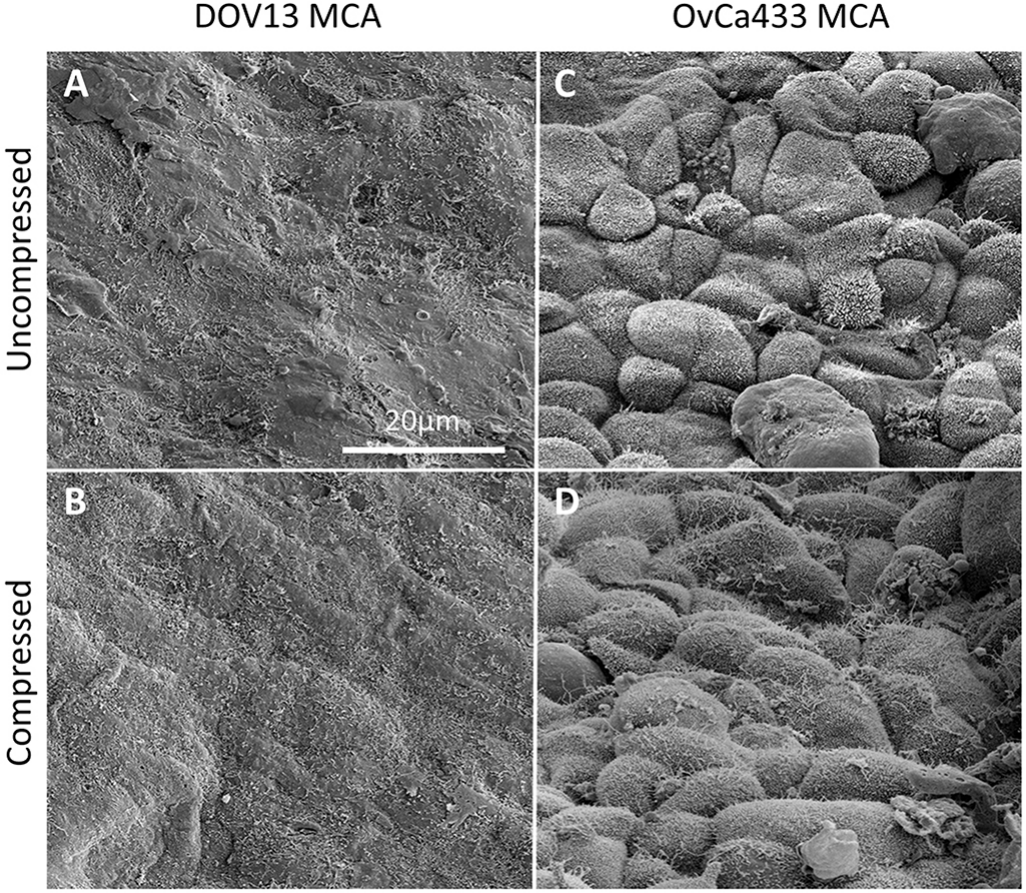
**Compression and MCA microarchitecture.** DOV13 and OvCa433 MCAs were subjected to compression using the Flexcell-4000C Compression Plus System for 24 h, processed for SEM, as detailed in the Materials and Methods, and examined using an FEI-Magellan 400-field emission scanning electron microscope. (A) Uncompressed DOV13 MCA. (B) Compressed DOV13 MCA. (C) Uncompressed OvCa433 MCA. (D) Compressed OvCa433 MCA. Representative images were taken at 5000× magnification. Scale bar: 20 μm.

We have previously reported that MCAs generated using the hanging drop method from N-cadherin-expressing cells form highly cohesive, smooth spheroids, whereas E-cadherin-expressing cells generate MCAs characterized by loose aggregates with uniform microvilli (Klymenko et al., 2017c). To determine whether MCA formation in the hydrogel wells and subsequent compression altered MCA morphology, MCA microarchitecture was examined using scanning electron microscopy (SEM). No significant compression-induced changes were observed in the microarchitecture of MCAs generated from either N-cadherin- or E-cadherin-expressing cells (Fig. 4A-D). Although enhanced cell-surface projections are apparent in compressed OvCa433 cells, the nature of these projections is unclear and is under further investigation.

**Fig. 5.**
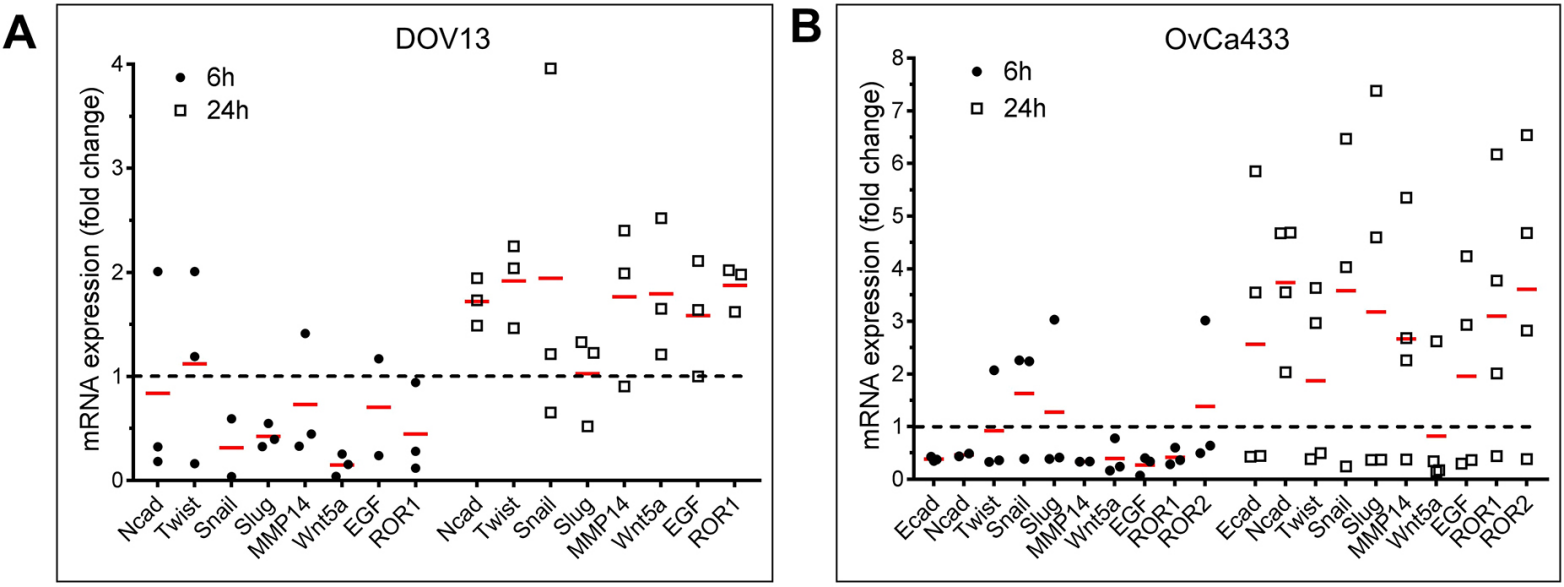
**Compression-induced changes in epithelial-mesenchymal-transition-associated genes.** (A) DOV13 and (B) OvCa433 MCAs were compressed employing a FX Flexcell-4000C Compression Plus System, and RNA was extracted and processed for qPCR as described in the Materials and Methods. Data shown are fold change in the expression of the indicated genes at 6 h (filled circles) and 24 h (open boxes), with red bars depicting the mean for each gene (representative of *n*≥3 experimental replicates, where each filled circle or open box is a separate replicate). Note that DOV13 cells do not express *ROR2* or *CDH1* (E-cadherin).

To evaluate the effect of compression on MCA gene expression, a panel of candidate genes associated with epithelial-mesenchymal transition was chosen for analysis by quantitative PCR (qPCR) (Table S1). Overall, short-term compression (6 h) downregulated gene expression, whereas compression for 24 h resulted in increased gene expression (Fig. 5A,B). Interestingly, in mesenchymal-type (N-cadherin^+^) DOV13 cells, *CDH2* (N-cadherin) mRNA expression was downregulated after short-term compression (6 h), and was accompanied by a consistent decrease in *SNAI1*, *SNAI2* (*SLUG*), *WNT5A*, *ROR1* and *ROR2* expression (Fig. 5A). After prolonged compression (24 h), however, mRNAs for *CDH2*, *TWIST*, *MMP14*, *WNT5A* and *ROR1* were stably elevated. In epithelial-type (E-cadherin^+^) cells, short-term compression also decreased gene expression, whereas compression for 24 h enhanced the expression of many genes, including *CDH2* (Fig. 5B).

**Fig. 6.**
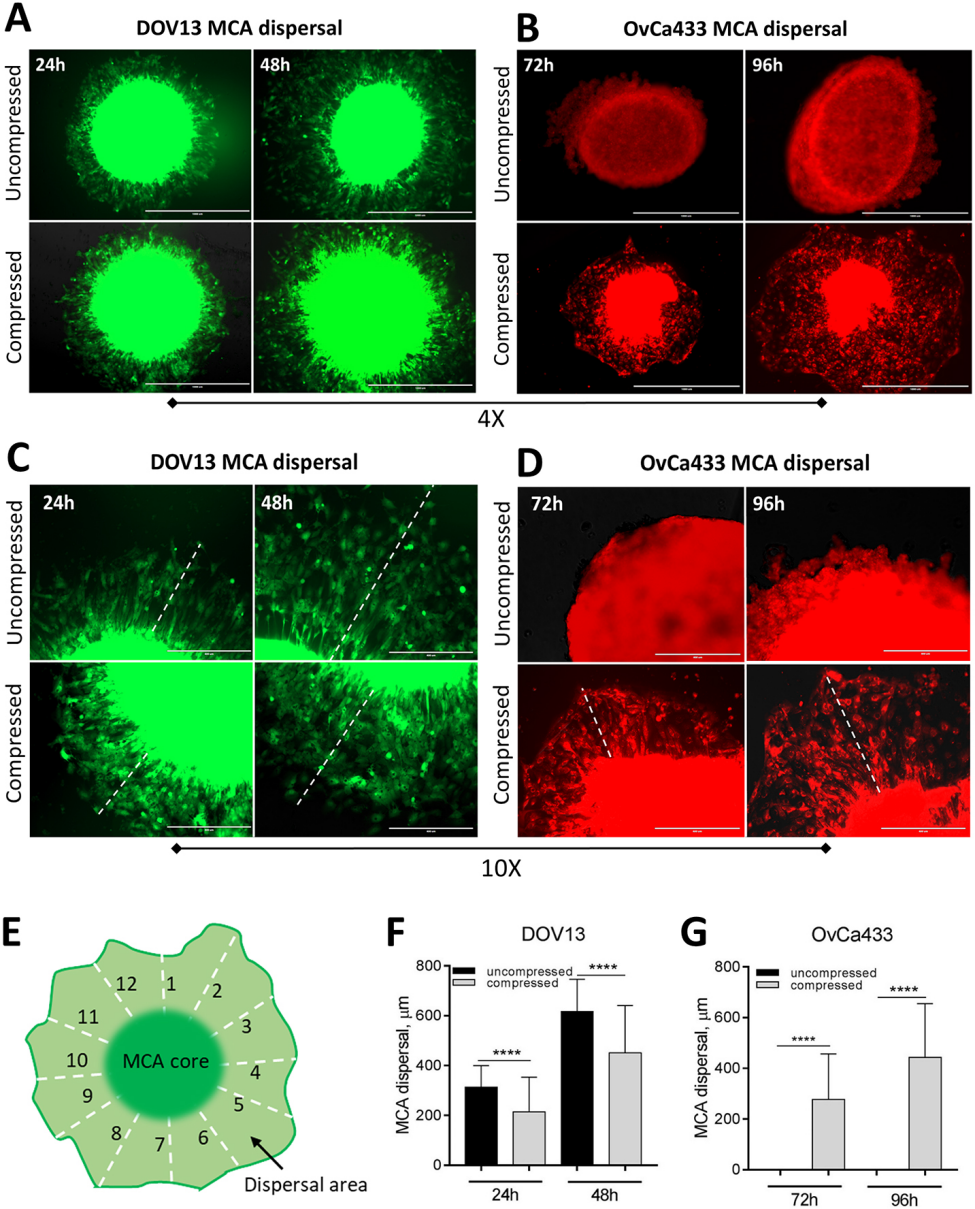
**Effect of compression on multicellular aggregate dispersal on collagen gels.** (A-D) Multicellular aggregates of DOV13-GFP (A,C) or OvCa433-RFP (B,D) cells were compressed for 48 h, removed from the hydrogel and seeded onto type I collagen gels in a 24-well plate. (E) Plates were photographed at 24, 48, 72 and 96 h, and the lateral dispersal of each individual MCA (*n*=15) for each time point was quantified in ImageJ by measuring the distance from the MCA core boundary over 12 radial lines uniformly distributed over the MCA dispersal area. Statistical significance (defined as *P*<0.05) was calculated using a two-sided Mann–Whitney *U* test. Control (uncompressed) MCAs were seeded into hydrogel wells and incubated under identical conditions but were maintained at atmospheric pressure. (F) Quantitation of DOV13 MCA dispersal. (G) Quantitation of OvCa433 MCA dispersal. ****P<0.0001. Scale bars: 1 mm (A,B), 400 μm (C,D).

During EOC metastasis, MCAs adhere to peritoneal mesothelial cells and submesothelial type I collagen, upon which they spread, induce mesothelial cell retraction, locally anchor within the collagen-rich submesothelial matrix and proliferate to generate secondary lesions (Lengyel et al., 2014; Klymenko et al., 2017b). To examine the potential impact of ascites-induced compression on downstream events in EOC metastasis, compressed or control MCAs generated with green fluorescent protein (GFP)-tagged DOV13 cells or red fluorescent protein (RFP)-tagged OvCa433 cells were removed from the hydrogel carrier, placed into collagen-coated wells and photographed for 0-96 h, followed by analysis of lateral dispersal area. Whereas a slight inhibition of DOV13 MCA dispersal was observed following compression (Fig. 6A,C,F), compression significantly enhanced lateral dispersal of OvCa433 MCAs (Fig. 6B,D,G).

## DISCUSSION

The unique transcoelomic route of EOC metastasis leads to obstruction of peritoneal lymphatic drainage (Holm-Nielsen, 1953; Feldman et al., 1972; Garrison et al., 1987). Together with enhanced tumor vascularization, secretion of factors such as vascular endothelial growth factor by tumor cells increases vascular permeability and thereby leads to the formation of a transudate with high protein concentration in the peritoneal cavity (Garrison et al., 1986). The accumulation of malignant ascites has been shown to induce dramatic (four- to fivefold) changes in intraperitoneal pressure (Parsons et al., 1996), altering intraperitoneal mechanobiology. In the current study, we report on the initial development of novel *in vitro* model systems with which to elucidate the potential influence of altered intraperitoneal pressure on EOC MCA structure, function and gene expression. Our initial results show that although proliferation is not affected, expression of genes associated with epithelial-mesenchymal transition as well as multicellular aggregate dispersal are differentially regulated by compression. During late-stage ovarian cancer metastasis, free-floating tumor cells and MCAs must acquire the ability to adhere to and anchor in the peritoneal cavity (Hudson et al., 2008). Our data suggest that compressive forces might facilitate metastasis by modulating cell-cell and cell-matrix adhesions rather than proliferative signaling pathways. This is consistent with data showing that solid (compressive) stresses inhibit mitosis in multicellular tumor spheroids (Desmaison et al., 2013) and tumor spheroid growth *in vitro* in human colon adenocarcinoma, murine mammary carcinoma and rat rhabdomyosarcoma cell lines (Helmlinger et al., 1997; Delarue et al., 2014; Stylianopoulos et al., 2013; Alessandri et al., 2013; Cheng et al., 2009).

Emerging data support the tenet that virtually all cells are capable of sensing mechanical changes in the extracellular *milieu* and initiating appropriate responses to maintain tensional homeostasis by converting biomechanical force into biochemical signals and cellular responses (Ingber, 1997, 2003, 2008; Butcher et al., 2009; Mui et al., 2016). Moreover, loss of tensional homeostasis in tissues can lead to phenotypic and genotypic modifications that can potentiate tumor progression. Cytoskeletal rearrangements occur as a response to mechanical cues, altering cell shape and thereby impacting tissue organization via regulation of integrin-cadherin crosstalk (Tse et al., 2012; Mui et al., 2016). Elevated interstitial fluid pressure as high as 50 mmHg has been reported in solid tumors (Piotrowski-Daspit et al., 2016), and numerous studies have linked elevated interstitial fluid pressure to phenotypic and genotypic changes that enhance malignancy and tumor progression (Farge, 2003; Koike et al., 2002; Piotrowski-Daspit et al., 2016; Lou et al., 2008).

Several studies have examined the effect of high interstitial fluid pressure on a variety of tumor cell types. For example, aggregates of MDA-MB-231 breast cancer or PC-3 prostate cancer cells embedded in collagen gels in polydimethylsiloxane chambers were subjected to a hydrostatic pressure gradient. Expression of genes associated with epithelial-mesenchymal transition as well as invasive activity were differentially regulated (Piotrowski-Daspit et al., 2016). Similar results were reported when stresses were >5.8 mmHg in studies using a weighted piston to press breast cancer cells against a membrane (Tse et al., 2012). In an alternative model, high-molecular-weight dextran was added to the culture medium of tumor spheroids in agarose to exert mechanical stress of 5-10 kPa. A decrease in spheroid volume, together with induction of p27^Kip1^ and inhibition of proliferation, was observed (Delarue et al., 2014).

In the current study, we have developed two novel methods to model the potential impact of compressive stress owing to accumulation of ascites on EOC multicellular aggregate cultures. Similar to previous reports, we show altered expression of genes associated with epithelial-mesenchymal transition. Interestingly, at early time points (6 h) gene expression was downregulated, whereas long-term compression (24 h) enhanced expression of a panel of genes related to epithelial-mesenchymal transition. These changes were observed in both E-cadherin-expressing OvCa433 cells with an epithelial phenotype as well as in mesenchymal N-cadherin-expressing DOV13 cells. Remarkably, early downregulation of both E-cadherin and N-cadherin as well as E-cadherin transcriptional repressors and other EMT mediators occurred concurrently. A possible explanation for this transient pervasive transcriptional downregulation might lie in the cellular adaptive response to the sudden intense compression, resulting in temporary transcriptional restriction of the majority of genes in favor of stress response mechanisms. In support of this hypothesis, multiple studies have reported activation of differential stress response patterns specific for distinct external biophysical stimuli. For example, a whole genome microarray hybridization study (Fernandes et al., 2004) using *Saccharomyces cerevisiae*, a classic model for assessment of cell stress responses, defined a hydrostatic pressure-specific stress response expression profile inclusive of more than twofold augmentation of stress defense and carbohydrate metabolism gene transcription, accompanied by downregulation of cell cycle progression and protein synthesis-associated genes. A stretch-specific gene activation signature has been described in biomechanically stretched cardiomyocytes with non-specific repression of the remaining genes (Frank et al., 2008). In a murine model of pressure overload-induced cardiac hypertrophy, an early and transitory restriction of genes responsible for energy metabolism occurs as an adaption to extreme pressure loading (van den Bosch et al., 2006), followed by subsequent differential ‘tuning’ of distinct pathways at 1, 5 and 8 weeks of continuous increased pressure.

Our results also show that compressed aggregates derived from the epithelial cell line OvCa433 were highly migratory, whereas control (uncompressed) multicellular aggregates did not disperse on collagen gels. Several laboratories have shown that, following initial tumor cell adhesion to the peritoneal mesothelial monolayer, metastasizing EOC cells induce rapid mesothelial cell retraction to expose the underlying 3D interstitial collagen matrix to which they avidly adhere, and into which they invade to anchor, proliferate and generate metastatic nodules (Niedbala et al., 1985; Davidowitz et al., 2012, 2014; Klymenko et al., 2017a). Under control conditions, OvCa433 multicellular aggregates do not readily invade 3D collagen gels; however, transfection with N-cadherin enhances invasive activity (Klymenko et al., 2017a). In the current study, using the Flexcell system following 24 h compression, increased N-cadherin gene expression was observed in OvCa433 cells, suggesting a potential transition towards a ‘hybrid cadherin’ phenotype (Klymenko et al., 2017c); however, additional experiments using longer-term compression are necessary to fully test this hypothesis. We have previously demonstrated that ‘hybrid cadherin’ EOC cells, in which E-cadherin and N-cadherin are expressed within the same cell, are significantly more adhesive to both organotypic mesomimetic cultures and to *ex vivo* peritoneal explants, with enhanced migration and matrix invasion. Furthermore, ‘hybrid cadherin’ expression patterns are found in human EOC tumors and cell lines (Klymenko et al., 2017c). Indeed, it is now well recognized that cancer cells can dynamically exist in a range of intermediate, or ‘metastable’, states (Jolly et al., 2015, 2016; Tam and Weinberg, 2013; Huang et al., 2013), which might provide a variety of advantages to the cells such as drug and radiation resistance (Jolly et al., 2015; Bastos et al., 2014; Hiscox et al., 2006; Wu et al., 2012). Together, these data suggest that enhanced intraperitoneal fluid pressure might contribute to the generation of the hybrid cadherin phenotype and thereby impact survival and implantation of metastatic MCAs. To this end, it is interesting to note a recent study demonstrating that ligation of N-cadherin was key to the ability of mesenchymal stem cells to sense alterations in matrix stiffness and regulate YAP/TAZ-based mechanosignaling (Cosgrove et al., 2016).

Ascites-induced increases in intraperitoneal pressure have negative implications at the clinical level. Development of tense ascites, usually associated with stage III or IV disease, is associated with severe discomfort, poor prognosis and fatality in women with EOC (Rahaman et al., 2004; Puls et al., 1996). Although the effects of soluble factors in ascites – including bioactive lipids, growth factors and cytokines – on peritoneal spreading have been studied in detail (Klymenko et al., 2017b), the role of ascites-induced changes in peritoneal mechanobiology has not been examined. The model systems described herein will have broad utility for future mechanistic analyses of compression-induced mechanotransduction and altered gene expression that impacts functional responses related to enhanced intraperitoneal dissemination.

## MATERIALS AND METHODS

### Cell culture

The EOC cell lines OvCa429, OvCa433 and DOV13 were kindly provided by Dr Robert Bast (University of Texas MD Anderson Cancer Center, Houston, TX, USA). All cells were maintained in minimal essential medium (Gibco, Big Cabin, OK, USA) containing 10% fetal bovine serum (FBS; Gibco), 1% non-essential amino acids (NEAA; Corning Cellgro, Manassas, VA, USA), 1% penicillin/streptomycin (Pen/Strep; Lonza, Allendale, NJ, USA), 1% sodium pyruvate (Corning Cellgro), 0.1% amphotericin B (Cellgro). DOV13 medium was additionally supplemented with 10 μg/ml insulin (Gibco). SKOV.3.ip cells were obtained from Dr Katherine Hale (University of Texas MD Anderson Cancer Center) and maintained in RPMI-1640 medium (Corning Cellgro), supplemented with 10% FBS, 1% L-glutamine (Gibco by Life Technologies), 1% sodium pyruvate, 1% Pen/Strep, 1% NEAA, 1% 4-(2-hydroxyethyl)-1-piperazineethanesulfonic acid (Gibco by Life Technologies) and 0.1% amphotericin B. Cells were incubated at 37°C in 5% CO_2_, routinely passaged upon achievement of 90-95% confluence and fed every 2-3 days. Cell lines were tested and authenticated by Genetica DNA Laboratories using short tandem repeat DNA profiling and were found to be >95% concordant. The cells tested negative for mycoplasma in 2015.

### Application of hydrostatic pressure to EOC MCAs

To mimic the increased peritoneal fluid pressure that accompanies tense ascites in women with ovarian cancer (Henriksen et al., 1980; Gotlieb et al., 1998), EOC cells were cultured as MCAs under conditions that model the peritoneal cavity as a fluid-filled sac. Cell suspensions (2×10^6^ cells/ml, 12 ml) were seeded into sterile low-density virgin polyethylene bags (Fisher Scientific, Hampton, NH, USA), containing complete culture medium appropriate for each cell line as indicated above, and sealed using a bag sealer (Fig. 1A). The bags were placed in 145×20 mm culture dishes (no lid), then placed in the incubator at 37°C, 5% CO_2_. Cells were allowed to form aggregates for 3 days (OvCa429, OvCa433) or 4 days (DOV13, SKOV.3.ip) then transferred to pressure vessels pre-heated to 37°C (Fig. 1B). Aggregates of varying sizes as well as single cells are observed (Fig. S1). Temperature was maintained at 37°C during the assay with a water bath incubator. The cylindrical pressure vessel had an inside diameter of 9.53 cm and was 12.9 cm deep, with a total volume 920 ml (Parr Instrument, Moline, IL, USA). Pressure was conveyed (0.5 pounds per square inch; 25 mmHg) to the test vessel using the Instron 88215, a servohydraulic testing system, via displacement of water in a medium-duty hydraulic pump (PHD, Fort Wayne, IN, USA) connected to the pressure vessel with stainless steel tubing; two valves were closed to contain the fluid pressure (Wagner et al., 2008; Steward et al., 2014). A control pressure vessel was sealed and incubated along-side the test pressure vessel at atmospheric pressure. After 8 h, bags were removed from the vessels and cells were harvested by decanting the MCA suspension into 15 ml conical tubes. Suspensions were processed for proliferation assays and western blotting as described below.

### Compression of EOC MCAs using the Flexcell Compression Plus System

To enable compression of MCAs under more controlled parameters, an FX Flexcell-4000C Compression Plus System (Flexcell International, Hillsborough, NC, USA) was used. Because the sample must physically touch the specimen platform and the adjustable platen, and to ensure even loading conditions, custom molds were fabricated to enable production of hydrogels to accommodate EOC MCAs and enable rapid recovery after compression for downstream analyses. Two molds were fabricated: a ‘lid’ mold to generate a spherical hydrogel with a 13 mm diameter and 1 mm height, and a ‘carrier’ mold with a 13 mm diameter and 2 mm height, designed to generate four circular voids for MCA placement. Prior to each experiment, the molds were cleaned, autoclaved and kept in sealed sterilizer bags. Molds were pre-warmed to 65°C for 10 min between hydrogel fabrication steps. Hydrogels were fabricated using UltraPure Agarose powder (2%, 15510-027, Invitrogen, Carlsbad, CA, USA) in phosphate-buffered saline (PBS; Corning Cellgro). Molten agarose solution was stored short-term in a 65°C oven at all times between hydrogel fabrication steps. Sterile and pre-warmed metal molds were immediately filled with 65°C molten agarose (220 µl into carrier molds; 165 µl into lid molds) and allowed to gel at room temperature (RT) for 10 min in a laminar flow hood (Fig. S2). Solidified hydrogels were removed from disassembled molds using a sterile spatula and placed into 70% ethanol until use. Hydrogels were then soaked in sterile PBS (1 h) and immersed into complete cell culture medium (30 min). Carrier hydrogels were placed into the foam sample holders of six-well BioPress culture plates with silicone elastomer well bottoms (Flexcell International Corporation, Hillsborough, NC, USA) immediately prior to cell seeding (Fig. S3). Carrier hydrogel wells (in BioPress plates) were seeded with pelleted cells (3 µl) using a 10 µl pipette tip and covered with the lid hydrogel. Fresh complete culture medium appropriate for each cell line as indicated above (3 ml) was added to each well of a BioPress plate, and encapsulated cells were incubated at 37°C in 5% CO_2_ for 48 h to enable MCA formation, with medium exchange after 24 h. BioPress plates with the MCAs/hydrogels were then filled with 4 ml of fresh culture medium, stationary platens were inserted into the culture plate wells (platen heights adjusted according to the specific parameters of individual hydrogels), and the compression baseplate was assembled according to the manufacturer's specifications. The MCAs were incubated at 37°C in 5% CO_2_ under static compression (3.18-3.53 kPa; ∼24-26.5 mmHg) applied to hydrogels for 6 or 24 h. Control MCAs were sealed in hydrogels, incubated in BioPress plates and placed in the same incubator, with no compression applied. A total of 24 compressed and 24 uncompressed hydrogels (four MCAs per hydrogel) were used for each biological experimental replicate. Immediately after termination of compression, plates were disassembled, hydrogels unsealed, MCAs harvested and stored in RNAlater (RO901, Sigma-Aldrich, St Louis, MO, USA) for mRNA extraction, or primary fixative solution for electron microscopy processing, as detailed below.

### Analysis of proliferation

Following termination of compression, proliferation was evaluated using a Cell Proliferation ELISA (Sigma-Aldrich) according to the manufacturer's specifications. Alternatively, MCAs were dispersed by incubation in 1 ml 0.25% trypsin for 10 min. Suspensions were diluted in 9 ml PBS, then counted using a Z2 Coulter Particle Count and Size Analyzer (Beckman Coulter, Brea, CA, USA). Assays were performed in triplicate and *P*-values were calculated by paired Student's *t*-test comparing compressed versus uncompressed cells.

### Western blot analysis

Compressed and control MCAs were incubated in modified radio-immunoprecipitation assay lysis buffer (mRIPA; 50 mmol/l Tris pH 7.5, 150 mmol/l NaCl, 0.1% SDS, 1% Triton X-100, 5 mmol/l EDTA)×3 h on ice and vortexed every 20-30 min. Protein concentrations of the resulting lysates were calculated with a DC protein assay kit (Bio-Rad, Hercules, CA, USA). Lysates (20 μg) were electrophoresed on an 9% SDS-polyacrylamide gel, transferred to a polyvinylidene difluoride membrane (Immobilon-P, EMD Millipore, Burlington, MA, USA) using a Bio-Rad Trans-Blot SD Semi-Dry Transfer Cell device, and blocked in 3% bovine serum albumin (BSA) in TBST buffer (25 mmol/l Tris pH 7.5, 150 mmol/l NaCl, 0.1% Tween 20) for 1 h at RT. Blots were incubated overnight with mouse monoclonal anti-N-cadherin (333900, Life Technologies, Carlsbad, CA, USA) or rabbit monoclonal anti-E-cadherin (ab40772, Abcam, Cambridge, UK) primary antibody, 1:1000 dilution, in 3% BSA/TBST overnight at 4°C, washed with TBST 3×5 min and further incubated with the peroxidase-conjugated goat anti-mouse (A4416, Sigma-Aldrich) or goat anti-rabbit (A6667, Sigma-Aldrich) immunoglobulin G secondary antibody, respectively, 1:4000 dilution, in 3% BSA/TBST for 30 min at RT. The antibody-tagged protein bands were developed with a SuperSignal West Dura chemiluminescent extended duration substrate kit (Thermo Fisher Scientific, Waltham, MA, USA) and visualized with an ImageQuant LAS4000 biomolecular imager (GE Healthcare, Chicago, IL, USA). For *GAPDH* housekeeping gene loading control confirmation, membranes were incubated in a 400 mmol/l glycine pH 2.5 stripping buffer for 30 min, blocked again in 3% BSA/TBST and re-probed with the peroxidase-conjugated mouse monoclonal anti-GAPDH antibody (G9295, Sigma-Aldrich), 1:25,000 dilution, in 3% BSA/TBST overnight at 4°C. Densitometric analysis of western blot protein bands was performed in ImageJ (https://imagej.nih.gov/ij/).

### RNA extraction and qPCR

Compressed and control MCAs were stored in RNAlater at 4°C, and RNA extraction was further performed with TRIzol LS Reagent (10296010, Ambion by Life Technologies). A QuantiTect Reverse Transcription Kit (Qiagen, Hilden, Germany) was employed to synthesize complementary DNA (cDNA) using 1 µg total RNA per reaction. For further amplification, 5 µl of a cDNA product in 20 µl volumes of iTaq Universal SYBR Green Supermix (Bio-Rad) and gene-specific primers at 200 nM final concentration each were used. PCR reactions were run on a Bio-Rad Thermal iCycler device using a five-cycle protocol according to the manufacturer's manual. The primer oligonucleotide pairs were obtained from Integrated DNA Technologies (Coralville, IA, USA). The full list of PCR target genes and their primer sequences is provided in Table S1. Relative quantification of mRNA expression was calculated according to the ΔΔCt method, with control (uncompressed) samples serving as a reference and ribosomal protein S13 (*RPS13*) housekeeping gene used as a normalizer. Data are presented as ‘fold change’ in mRNA expression, defined as the ratio of expression in compressed relative to uncompressed cells.

### SEM

Compressed and control MCAs were immersed in primary fixative solution (2% glutaraldehyde, 2% paraformaldehyde in 0.1 M cacodylate buffer pH 7.35) and rotated overnight at 4°C in Eppendorf tubes, followed by 3×20 min washes in 0.1 cacodylate buffer. Secondary processing was performed with 2% osmium tetroxide in 0.1 cacodylate buffer using a PELCO^®^ EM Pro Microwave vacuum chamber (TED PELLA, Redding, CA, USA). Samples were then washed with ultrapure water 3×5 min, dehydrated in a series of increasing ethanol concentrations (20%, 50%, 70%, 90%, 3×100%), followed by critical point drying using Tousimis-931 dryer, placed on carbon stubs, mounted with colloidal silver liquid (TED PELLA), coated with iridium (108 Auto Sputter coater, TED PELLA) and examined under a FEI-Magellan 400-field emission scanning electron microscope (FEI, Hillsboro, OR, USA).

### MCA dispersal on matrix

DOV13 and OvCa433 cells were tagged with GFP and RFP, respectively, as previously described (Klymenko et al., 2017a). DOV13-GFP and OvCa433-RFP control and compressed (48 h) MCAs were carefully removed from the unsealed hydrogels using a sterile spatula and placed into wells of a 24-well plate pre-coated with rat tail collagen type I (RTCI, Corning; 10 µg/ml in 0.1 M sodium carbonate pH 9.6), one MCA per well, in fresh full medium. MCAs (*n*=15 per each condition) were incubated at 37°C in 5% CO_2_, and photographed using an EVOS fluorescence microscope (EVOS FL Cell Imaging System, Thermo Fisher Scientific, Waltham, MA, USA) at 24, 48, 72 and 96 h. The lateral dispersal of each individual MCA for each time point was quantified in ImageJ by measuring the distance from the MCA core boundary over 12 radial lines uniformly distributed over the MCA dispersal area. Statistical significance (defined as *P*<0.05) was calculated using a two-sided Mann–Whitney *U* test.

## Supplementary Material

Supplementary information

First Person interview
