# Supplementary material for: Modeling the effect of ascites-induced compression on ovarian cancer multicellular aggregates
Source: Dis Model Mech. 2018 Sep 25;11(9):dmm034199. doi: 10.1242/dmm.034199 (PMC6176988; doi:10.1242/dmm.034199)
Supplement: Supplementary information [file dmm-11-034199-s1.pdf]

SUPPLEMENTAL FIGURES

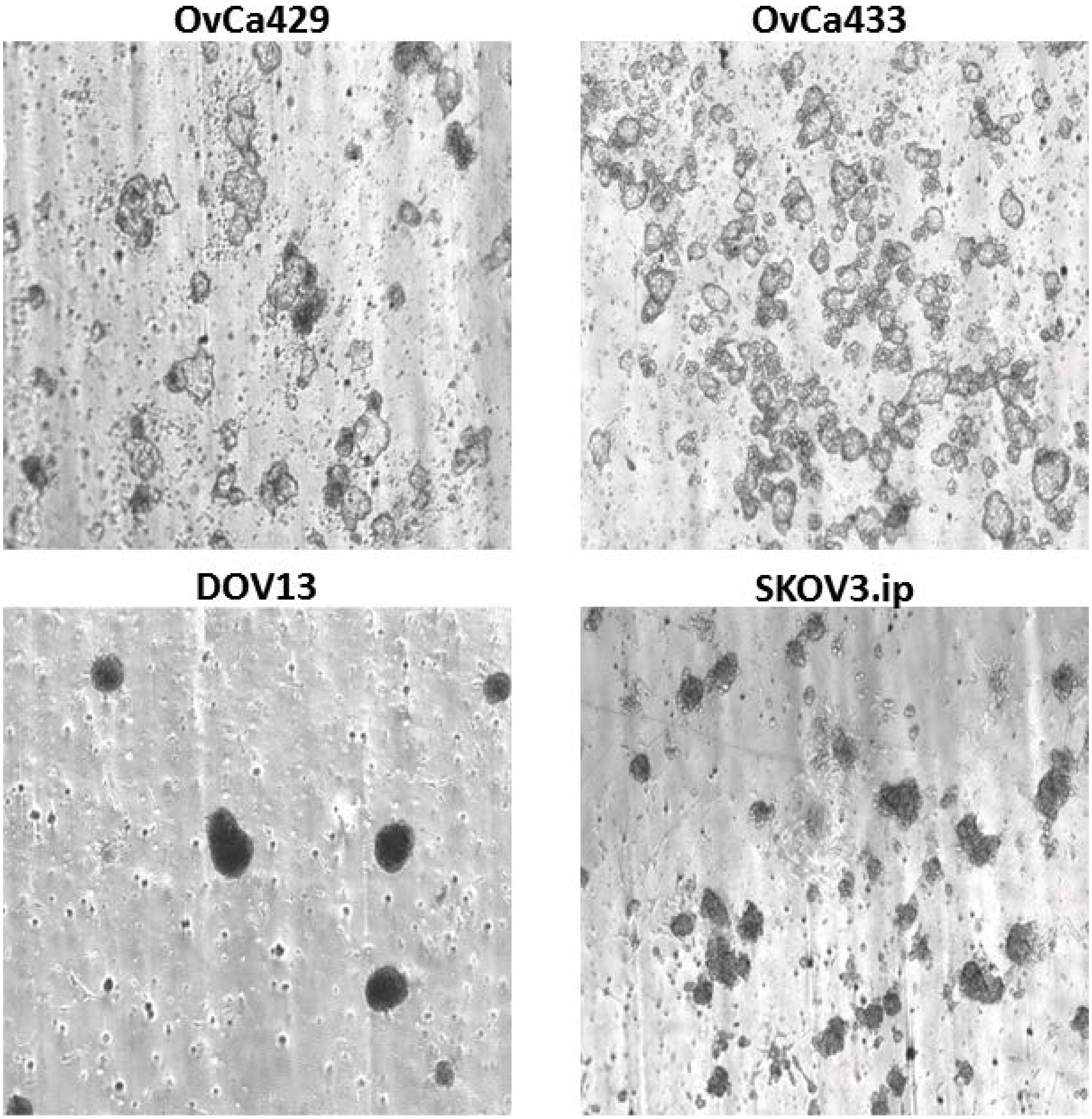

Fig. S1. Preparation of cells for Instron-based compression. Cells (2 x 10^6/ml) were seeded in supplemented MEM (OVCA429, OVCA433, DOV13) or RPMI (SKOV.3.ip), placed in an incubator (37°C, 5% CO_2) and allowed to form MCAs for 3 days (OVCA429, OVCA433) or 4 days (DOV13, SKOV.3.ip). Cells grown in bags were imaged using an EVOS FL Cell Imaging System (Thermo Fisher Scientific; brightfield, 20X).

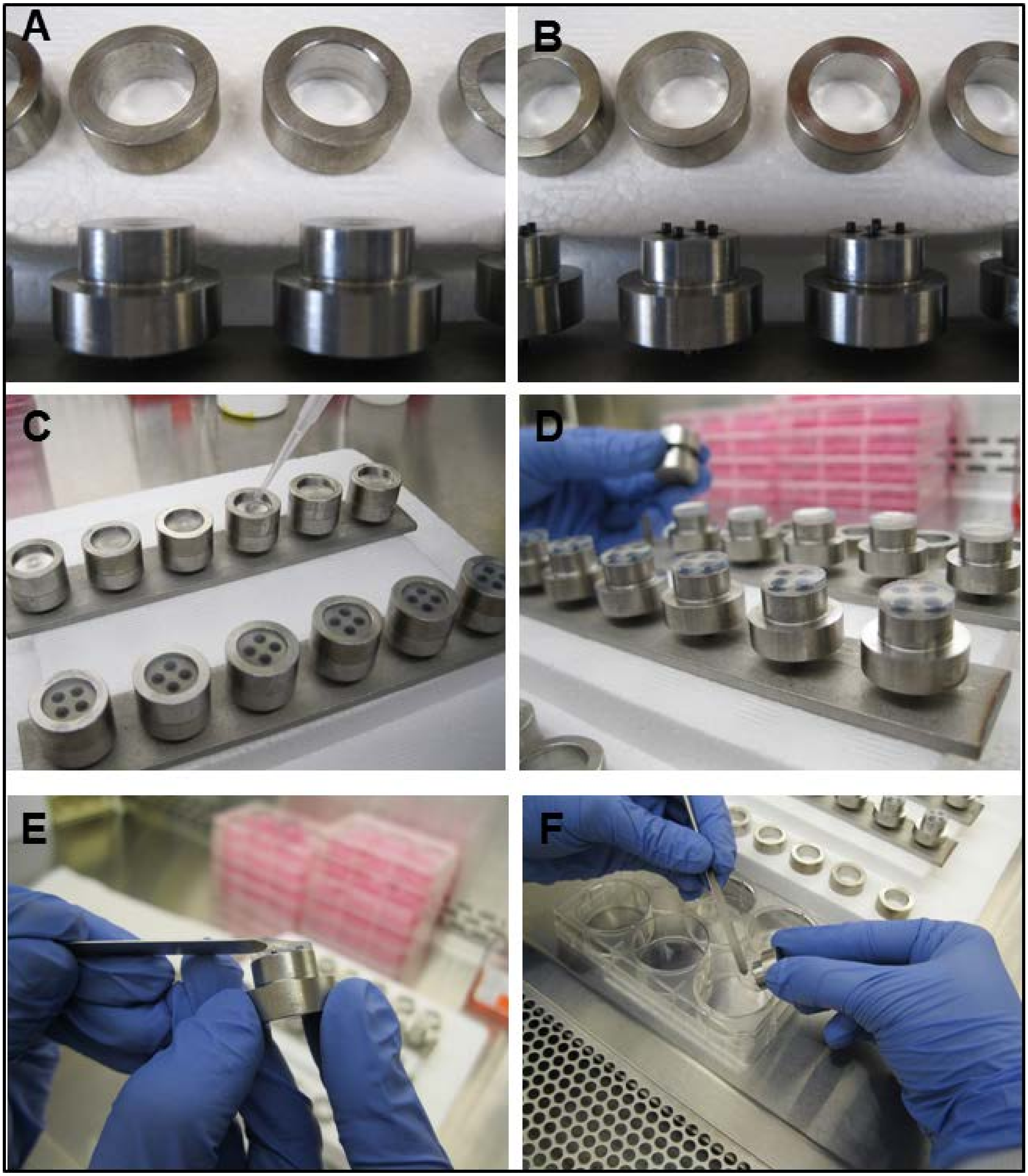

Disease Models & Mechanisms • Supplementary information

Fig. S2. Preparation of hydrogel carriers for EOC multicellular aggregates (MCAs). Custom molds were fabricated to enable production of hydrogels to accommodate EOC MCAs and enable rapid recovery after compression for downstream analyses. **(A)** A "lid" mold was designed to generate a spherical hydrogel with a 13 mm diameter and 1 mm height while **(B)** a "carrier" mold with a 13 mm diameter and 2 mm height designed to generate 4 circular voids for MCA placement. **(C,D)** Sterile and pre-warmed metal molds were filled with 65°C molten agarose (220µl – into carrier molds, 165µl – into lid molds) and allowed to gel at room temperature for 10 min in a laminar flow hood. **(E, F)** Solidified hydrogels were removed from disassembled molds using a sterile spatula and placed into 70% Ethanol until use. Hydrogels were then soaked in sterile PBS (1 hour) and immersed into complete cell culture medium (30 min).

Disease Models & Mechanisms • Supplementary information

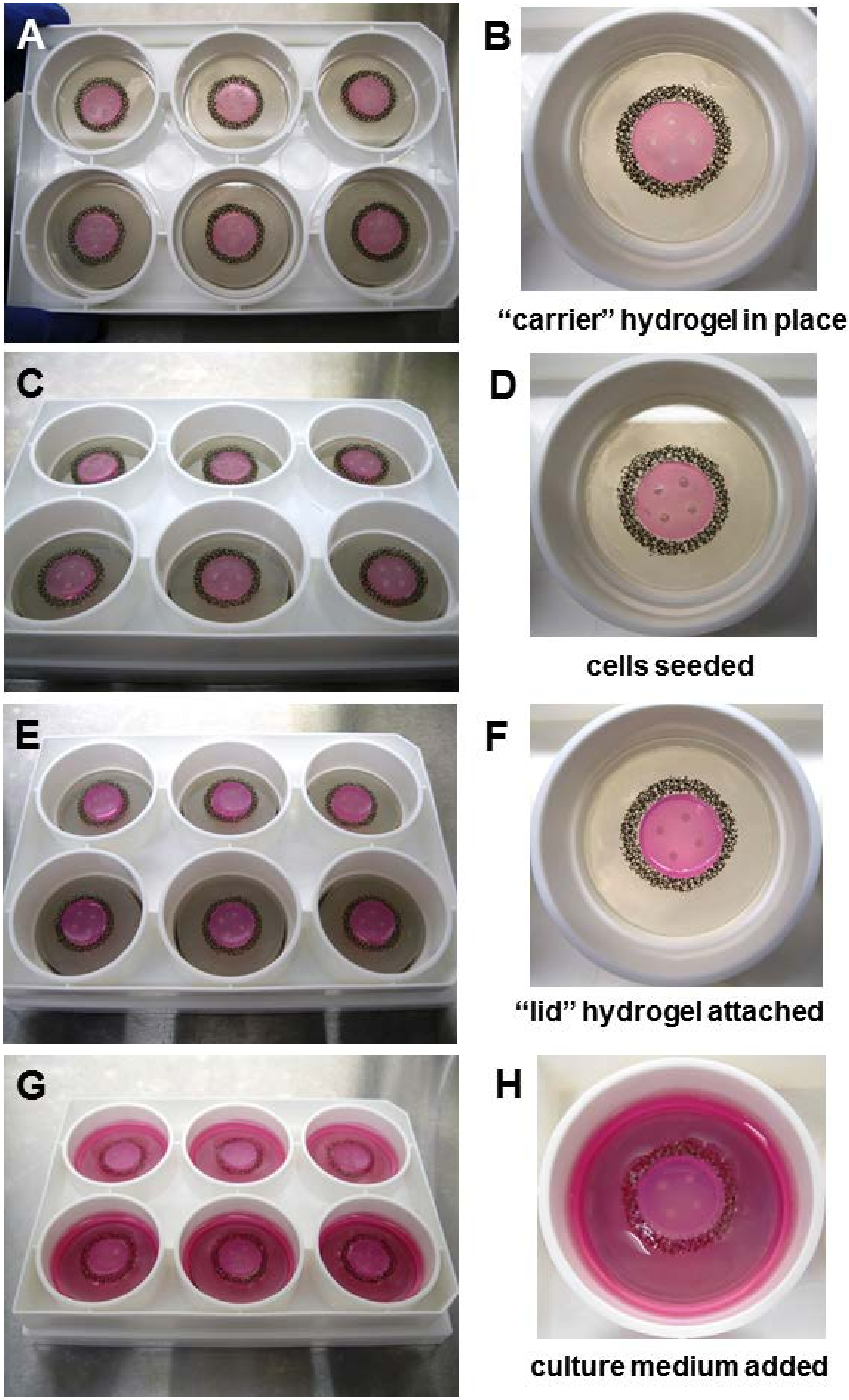

Disease Models & Mechanisms • Supplementary information

Fig. S3. Assembly of hydrogel carriers and EOC MCAs into compression plates. (A, B) Carrier hydrogels were placed into the foam sample holders of silicone elastomer bottom 6-well BioPressculture plates immediately prior to cell seeding. **(C-F)** Carrier hydrogel wells were seeded with cells and covered with the lid hydrogel. **(G,H)** Fresh medium (3 ml) was added to each well of the BioPress plate, and encapsulated cells were incubated at 37˚C in 5% CO_2 for 48 hours to enable MCAs formation, with medium exchange after 24 hours.

Table S1. qPCR target genes and primer sequences:

Gene Symbol	Approved Name (by HGNC*)	Properties	Primer Sequence	Primer Sequence Reference
CDH1	Cadherin 1, E-cadherin (Ecad)	Cell adhesion molecule, non-invasive epithelial cell phenotype	F: 5’- GCCAAGCAGCAGTACATTCTACACG -3’<br>R: 5’- GCTGTTCTTCACGTGCTCAAAATCC -3’	Matsuyoshi *et al.(1)*
CDH2	Cadherin 2, N-cadherin (Ncad)	Cell adhesion molecule, more invasive mesenchymal cell phenotype	F: 5’- GTGCCATTAGCCAAGGGAATTCAGC -3’<br>R: 5’- GCGTTCCTGTTCCACTCATAGGAGG -3’	Matsuyoshi *et al.(1)*
SNAI1	Snail family transcriptional repressor 1	Repressor of Ecad (2-8)	F: 5’- TCGGAAGCCTAACTACAGCGA -3’<br>R: 5’- AGATGAGCATTGGCAGCGAG -3’	Used in multiple studies
SNAI2 (SLUG)	Snail family transcriptional repressor 2	Repressor of Ecad (2, 9, 10)	F: 5’- CGAACTGGACACACATACAGTG-3’<br>R: 5’- CTGAGGATCTCTGGTTGTGGT -3’	Used in multiple studies
TWIST	Twist family bHLH transcription factor	Repressor of Ecad (5, 11-14)	F: 5'- CTCACGAGCGGCTCAGCTAC-3',<br>R: 5'- CTGGAAACAATGACATCTAGGTC -3’	Used in multiple studies
MMP9	Matrix metallopeptidase (metalloproteinase) 9	Cleavage of a functional 90 kDa Ncad ectodomain fragment => ↑ angiogenesis, Cleavage of Ecad ectodomain => more invasive cell phenotype (15-18)	F: 5’- CTTTGACAGCGACAAGAAGTGG -3’<br>R: 5’- TTCAGGGCGAGGACCATAGAG -3’	Used in multiple studies
MMP14	Matrix metallopeptidase (metalloproteinase) 14		F: 5’- CACTGCCTACGAGAGGAAGG -3’,<br>R: 5’- GAGCAGCATCAATCTTGTCG -3’	Used in multiple studies
EGF	Epidermal growth factor	Induction of Ecad to Ncad switch (15, 19, 20)	F: 5’- GCGTTCCTCTTAGCCCAGTA -3’<br>R: 5’- AATGGTTGTGGTCCTGAAGC -3’	Anelli *et al.(21)*
HGF	Hepatocyte growth factor	Induction of Ecad to Ncad switch (15, 22)	F: 5’- GGACAAGAACATGGAAGACT -3’<br>R: 5’- ACAACGAGAAATAGGGCAAT -3’	Lederer *et al.(23)*
WNT5A	Wingless-type MMTV integration site family, member 5A	Stimulation or suppression of EMT? Controversial data (24)	F: 5’- AGGGCTCCTACGAGAGTGCT -3’<br>R: 5’- GACACCCCATGGCACTTG -3’	Used in multiple studies
ROR1	Receptor tyrosine kinase-Like orphan receptor 1	Present in ovarian cancer stem cells, promotes EMT, migration/invasion, and chemoresistance; positive feedback loop with Wnt5a (25-29)	F: 5’- ACCGCACCGTGTATATGGAGTCT -3’<br>R: 5’- GCATAGTGGCACAGGGAAGG -3’	Used in multiple studies
ROR2	Receptor tyrosine kinase-Like orphan receptor 2		F: 5’- GGCAGAACCCATCCTCGTG -3’<br>R: 5’- CGACTGCGAATCCAGGACC -3’	Used in multiple studies
RPS13	Ribosomal protein S13	Housekeeping gene for renormalization	F: 5‘- CGAAAGCATCTTGAGAGGAACA -3‘<br>R: 5‘- TCGAGCCAAACGGTGAATC -3'	Jacob *et al.(30)*

* HUGO Gene Nomenclature Committee

Disease Models & Mechanisms • Supplementary information

References:

1. Matsuyoshi N, Toda K, Imamura S. N-cadherin expression in human adult T-cell leukemia cell line. Arch Dermatol Res. 1998;290(4):223-5.

2. Moreno-Bueno G, Cubillo E, Sarrio D, Peinado H, Rodriguez-Pinilla SM, Villa S, et al. Genetic profiling of epithelial cells expressing E-cadherin repressors reveals a distinct role for Snail, Slug, and E47 factors in epithelial-mesenchymal transition. Cancer Res. 2006 Oct 1;66(19):9543-56.

3. Blechschmidt K, Sassen S, Schmalfeldt B, Schuster T, Höfler H, Becker K. The E-cadherin repressor Snail is associated with lower overall survival of ovarian cancer patients. Br J Cancer. 2008;98(2):489-95.

4. Cano A, Pérez-Moreno MA, Rodrigo I, Locascio A, Blanco MJ, del Barrio MG, et al. The transcription factor snail controls epithelial–mesenchymal transitions by repressing E-cadherin expression. Nat Cell Biol. 2000;2(2):76-83.

5. Montserrat N, Gallardo A, Escuin D, Catasus L, Prat J, Gutiérrez-Avignó FJ, et al. Repression of E-cadherin by SNAIL, ZEB1, and TWIST in invasive ductal carcinomas of the breast: a cooperative effort? Hum Pathol. 2011;42(1):103-10.

6. Jiao W, Miyazaki K, Kitajima Y. Inverse correlation between E-cadherin and Snail expression in hepatocellular carcinoma cell lines in vitro and in vivo. Br J Cancer. 2002;86(1):98-101.

7. Rosivatz E, Becker K, Kremmer E, Schott C, Blechschmidt K, Höfler H, et al. Expression and nuclear localization of Snail, an E-cadherin repressor, in adenocarcinomas of the upper gastrointestinal tract. Virchows Archiv. 2006;448(3):277-87.

8. Yokoyama K, Kamata N, Hayashi E, Hoteiya T, Ueda N, Fujimoto R, et al. Reverse correlation of E-cadherin and snail expression in oral squamous cell carcinoma cells in vitro. Oral Oncol. 2001;37(1):65-71.

9. Hajra KM, Chen DY, Fearon ER. The SLUG zinc-finger protein represses E-cadherin in breast cancer. Cancer Res. 2002 Mar 15;62(6):1613-8.

Disease Models & Mechanisms • Supplementary information

10. Heuberger J, Birchmeier W. Interplay of cadherin-mediated cell adhesion and canonical Wnt signaling. Cold Spring Harb Perspect Biol. 2010 Feb;2(2):a002915.

11. Vesuna F, van Diest P, Chen JH, Raman V. Twist is a transcriptional repressor of E-cadherin gene expression in breast cancer. Biochem Biophys Res Commun. 2008;367(2):235-41.

12. Wang W, Yu S, Yang X, Chang S, Hou J. Expression and significance of twist and E-cadherin in ovarian cancer tissues. Asian Pacific Journal of Cancer Prevention. 2013;14(2):669-72.

13. Zhang Z, Xie D, Li X, Wong Y, Xin D, Guan X, et al. Significance of TWIST expression and its association with E-cadherin in bladder cancer. Hum Pathol. 2007;38(4):598-606.

14. Sasaki K, Natsugoe S, Ishigami S, Matsumoto M, Okumura H, Setoyama T, et al. Significance of Twist expression and its association with E-cadherin in esophageal squamous cell carcinoma. Journal of Experimental & Clinical Cancer Research. 2009;28(1):1.

15. Derycke LD, Bracke ME. N-cadherin in the spotlight of cell-cell adhesion, differentiation, embryogenesis, invasion and signalling. Int J Dev Biol. 2004;48:463-76.

16. Egeblad M, Werb Z. New functions for the matrix metalloproteinases in cancer progression. Nature Reviews Cancer. 2002;2(3):161-74.

17. Paradies N, Grunwald G. Purification and characterization of NCAD90, a soluble endogenous form of N-cadherin, which is generated by proteolysis during retinal development and retains adhesive and neurite-promoting function. J Neurosci Res. 1993;36(1):33-45.

18. Dwivedi A, Slater SC, George SJ. MMP-9 and -12 cause N-cadherin shedding and thereby beta-catenin signalling and vascular smooth muscle cell proliferation. Cardiovasc Res. 2009 Jan 1;81(1):178-86.

19. Cai Z, Wang Q, Zhou Y, Zheng L, Chiu J, He Q. Epidermal growth factor-induced epithelial–mesenchymal transition in human esophageal carcinoma cells–A model for the study of metastasis. Cancer Lett. 2010;296(1):88-95.

20. Ackland ML, Newgreen DF, Fridman M, Waltham MC, Arvanitis A, Minichiello J, et al. Epidermal growth factor-induced epithelio-mesenchymal transition in human breast carcinoma cells. Laboratory investigation. 2003;83(3):435-48.

21. Anelli L, Zagaria A, Coccaro N, Tota G, Impera L, Minervini CF, et al. A novel t(4;16)(q25;q23.1) associated with EGF and ELOVL6 deregulation in acute myeloid leukemia. Gene. 2013 10/15;529(1):144-7.

22. DeLuca SM, Gerhart J, Cochran E, Simak E, Blitz J, Mattiacci-Paessler M, et al. Hepatocyte growth factor/scatter factor promotes a switch from E-to N-cadherin in chick embryo epiblast cells. Exp Cell Res. 1999;251(1):3-15.

23. Lederer A, Herrmann P, Seehofer D, Dietel M, Pratschke J, Schlag P, et al. Metastasis-associated in colon cancer 1 is an independent prognostic biomarker for survival in klatskin tumor patients. Hepatology. 2015;62(3):841-50.

24. Asem MS, Buechler S, Wates RB, Miller DL, Stack MS. Wnt5a Signaling in Cancer. Cancers. 2016;8(9):79.

25. Henry C, Llamosas E, Djordjevic A, Hacker N, Ford C. Migration and invasion is inhibited by silencing ROR1 and ROR2 in chemoresistant ovarian cancer. Oncogenesis. 2016;5(5):e226.

26. Henry CE, Llamosas E, Hacker NF, Heinzelmann-Schwarz V, Ford CE. The role of the ROR receptors in ovarian cancer progression and chemoresistance. Cancer Res. 2016;76(14 Supplement):1636-.

27. Li X, Yamagata K, Nishita M, Endo M, Arfian N, Rikitake Y, et al. Activation of Wnt5a-Ror2 signaling associated with epithelial-to-mesenchymal transition of tubular epithelial cells during renal fibrosis. Genes to Cells. 2013;18(7):608-19.

28. Ren D, Minami Y, Nishita M. Critical role of Wnt5a–Ror2 signaling in motility and invasiveness of carcinoma cells following Snail-mediated epithelial–mesenchymal transition. Genes to Cells. 2011;16(3):304-15.

29. Tan H, He Q, Gong G, Wang Y, Li J, Wang J, et al. miR-382 inhibits migration and invasion by targeting ROR1 through regulating EMT in ovarian cancer. Int J Oncol. 2016;48(1):181-90.

30. Jacob F, Guertler R, Naim S, Nixdorf S, Fedier A, Hacker NF, et al. Careful selection of reference genes is required for reliable performance of RT-qPCR in human normal and cancer cell lines. PloS one. 2013;8(3):e59180.
