# Supplementary material for: Modeling the effect of ascites-induced compression on ovarian cancer multicellular aggregates
Source: Dis Model Mech. 2018 Sep 25;11(9):dmm034199. doi: 10.1242/dmm.034199 (PMC6176988; doi:10.1242/dmm.034199)
Supplement: First Person interview [file dmm-11-034199-s2.pdf]

FIRST PERSON

# First person – Yuliya Klymenko and Rebecca Wates

First Person is a series of interviews with the first authors of a selection of papers published in Disease Models & Mechanisms, helping early-career researchers promote themselves alongside their papers. Yuliya Klymenko and Rebecca Wates are co-first authors on 'Modeling the effect of ascites-induced compression on ovarian cancer multicellular aggregates', published in DMM. Yuliya is a Postdoctoral Research Associate in the lab of Dr Kenneth P. Nephew at Indiana University School of Medicine, IN, USA, and Visiting Research Scholar at the University of Notre Dame, Harper Cancer Research Institute, IN, USA, investigating genetic and epigenetic molecular mechanisms contributing to ovarian cancer metastatic progression, chemoresistance and recurrence. Rebecca is a Senior Scientist in the lab of Dr Andrew K. Godwin at University of Kansas Medical Center, KS, USA, working on a translational research project to develop novel therapeutics for the treatment of ovarian cancers and pediatric sarcomas.

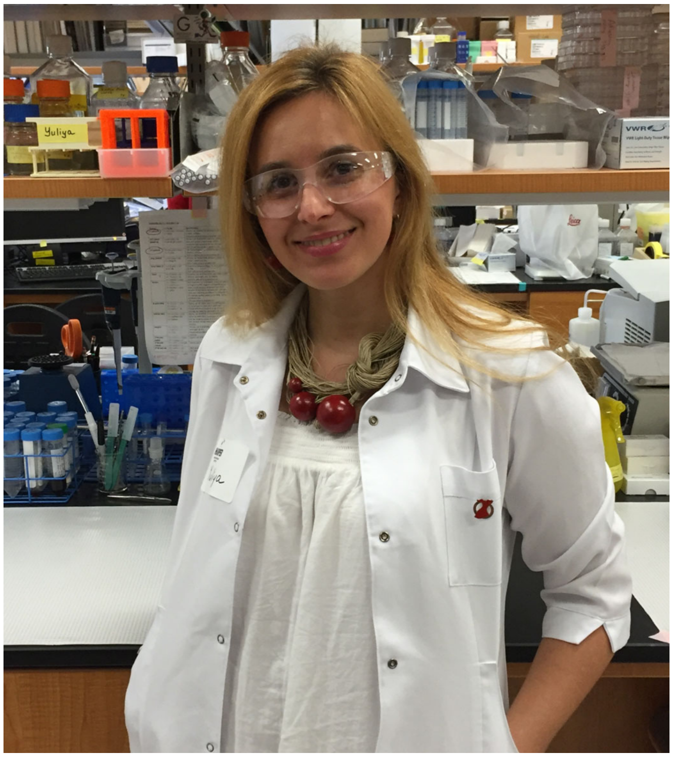

Yuliya Klymenko

**How would you explain the main findings of your paper to non-scientific family and friends?**

YK: Accumulation of large volumes of malignant fluid (ascites) in the abdominal cavity often accompanies ovarian cancer progression and significantly worsens patients' prognosis. In this paper, we present a novel methodology for controllable application of clinically relevant mechanical stress on ovarian cancer cell clusters that allows investigation of their molecular and functional characteristics under ascites-mimicked conditions in cell culture.

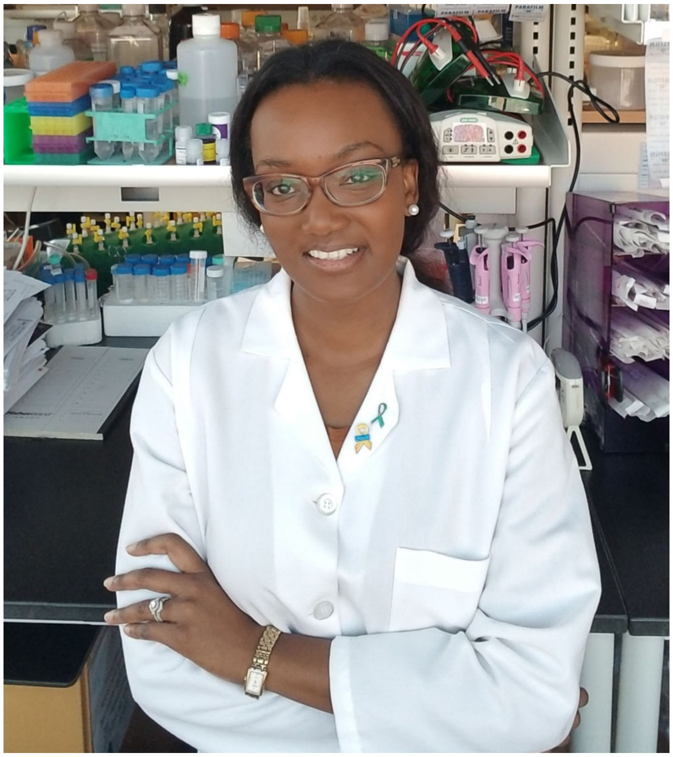

Rebecca Wates

RW: There are two exciting findings in this study: 1) we are the first to develop a method that reproduces the type and magnitude of compressive force exerted on spreading ovarian tumor cells; 2) we have shown that compressive forces can affect spreading cancer cells, causing them to acquire changes that make it easier for the cells to spread and possibly to avoid therapy-induced cell death.

**What are the potential implications of these results for your field of research?**

YK: Our developed compression systems will allow researchers to approach experimental evaluation of the impact of tumor-associated transudate accumulation and altered tumor mechanobiology, characteristic of ovarian and other cancer types (e.g. pancreatic and lung cancers).

RW: The field of mechanobiology is steadily developing, yet the full implications of how the mechanome impacts cell, tissue and whole-organism physiology remains unclear. Our work contributes to this burgeoning field, and importantly demonstrates that seemingly small changes in compressive force are sufficient to modulate changes in tumor cell signaling.

Yuliya Klymenko's contact details: Indiana University School of Medicine, Bloomington, IN 47405, USA
Rebecca Wates' contact details: Department of Pathology and Laboratory Medicine, University of Kansas Medical Center, Kansas City, KS 66160, USA.
E-mails: yklymenk@nd.edu; yklymenk@iu.edu; rwates@kumc.edu

**What are the main advantages and drawbacks of the model system you have used as it relates to the disease you are investigating?**

YK and RW: Our model allows for the controlled and systematic investigation of compressive forces; however, while the simplicity of the model allows us to isolate effects of compression it is not currently designed to elucidate the interplay between mechanical forces and other microenvironmental factors (e.g. immune responses). Also, the current model does not involve evaluation of other ascites-associated mechanical strain components, such as tissue tension and flow disturbances, which must be assessed separately.

**What has surprised you the most while conducting your research?**

> "I did not anticipate that such small changes in hydrostatic pressure would be sufficient to alter cellular signaling."

YK: To validate our compression system's feasibility we chose several EMT-related genes based on published literature, hoping to be lucky enough to observe altered expression in at least one or a few genes at first attempt and identify candidacy gene(s) for further research. It was quite intriguing and very promising to see such an avid response to applied compression in the vast majority of genes from our list.

RW: When I started this project using the 'cells-in-bags' model, I was uncertain that I would observe significant changes because I did not anticipate that such small changes in hydrostatic pressure would be sufficient to alter cellular signaling. I am excited that we have proved my uncertainties wrong!

**Describe what you think is the most significant challenge impacting your research at this time and how will this be addressed over the next 10 years?**

> "The good news is that significant advances in technical tools, rapid emergence of large 'omics' data and the ability to perform quick data analysis in large patient cohorts has vastly expanded current knowledge in the field."

YK: The big challenge is that the disease we generally call 'epithelial ovarian cancer' is a histopathologically, morphologically and molecularly heterogeneous group of neoplasms, and researchers' attempts to target certain ovarian cancer cell vulnerabilities have had limited effect. Even the origin of ovarian cancer is a matter of constant debate, as it is now known that at least half of ovarian carcinomas do not arise from ovarian tissue. The good news is that significant advances in technical tools, rapid emergence of large 'omics' data and the ability to perform quick data analysis in large patient cohorts has vastly expanded current knowledge in the field. In my opinion, an interdisciplinary collaborative effort between molecular biologists, geneticists, clinical oncologists, chemists, engineers, bioinformaticians and computational biology experts will define progress in translating this knowledge into the clinic.

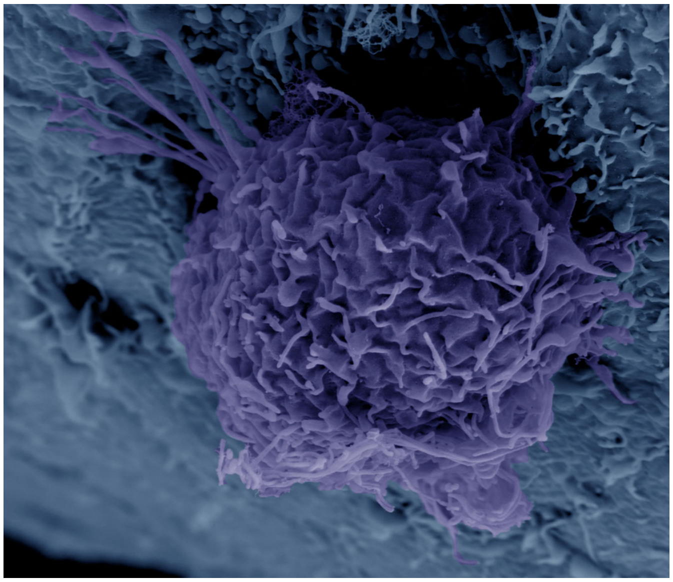

Pseudo-colored scanning electron micrograph of a metastasizing ovarian cancer cell (purple) attaching to the surface of a cancerous multicellular aggregate (blue). Image credit: Yuliya Klymenko.

RW: The ever-evolving technologies being employed in research create a lot of big data that may hold important answers waiting to be revealed. This fact, coupled with a need to rethink research spending to maximize limited grant funds, will push the trend for biologists to receive more formal education in biostatistics, so we can fully interrogate existing data.

**What changes do you think could improve the professional lives of early-career scientists?**

YK: Fear of career uncertainty, constant striving for research funds (with unguaranteed success), and the pressure of consistent, highly prolific publication productivity at the expense of a work-life balance makes many talented junior researchers choose less passionate but financially more secure and steady employment. Creating funding opportunities committed to embracing more grant proposals from early-career researchers, support of networking programs and interdisciplinary collaborations with well-established senior investigators will encourage young scientists' research careers.

RW: I personally believe that a lack of competitive salary and benefits (especially parental leave and day care options), coupled with few available faculty positions, are challenges that often affect young scientists' desire to remain in academic research positions. Ensuring financial compensation commensurate with training and experience and ensuring appropriate family support for early-career scientists will improve retention of young investigators and foster greater focus on advancing research.

**What's next for you?**

YK: At my current postdoctoral position, I was recently awarded the Indiana Clinical and Translational Sciences Institute Grant to pursue a project on epigenetic regulation of ovarian cancer progression and drug resistance. This will be the primary focus of my research for the next 1-2 years.

RW: I have identified a novel therapeutic target in ovarian cancers, and am currently working on a collaborative team to develop compounds that can be used in preclinical testing. I recently

completed my postdoctoral training as an American Cancer Society fellow, and I am now looking for faculty positions to continue my research in ovarian cancer and to advance new research in pediatric sarcomas.

**Reference**

**Klymenko, Y., Wates, R. B., Weiss-Bilka, H., Lombard, R., Liu, Y., Campbell, L., Kim, O., Wagner, D., Ravosa, M. J. and Stack, M. S.** (2018). Modeling the effect of ascites-induced compression on ovarian cancer multicellular aggregates. *Dis. Model. Mech.* **11**: dmm034199.
